# Knowledge, access and utilization of bed-nets among stable and seasonal migrants in an artemisinin resistance containment area of Myanmar

**DOI:** 10.1186/s40249-017-0353-8

**Published:** 2017-09-14

**Authors:** Wint Phyo Than, Tin Oo, Khin Thet Wai, Aung Thi, Philip Owiti, Binay Kumar, Hemant Deepak Shewade, Rony Zachariah

**Affiliations:** 1 Regional Public Health Department, Ministry of Health, Bago, Myanmar; 2 Department of Medical Research, Ministry of Health, Yangon, Myanmar; 3 National Malaria Control Program, Ministry of Health, Naypyitaw, Myanmar; 4 Academic Model Providing Access to Healthcare (AMPATH), Eldoret, Kenya; 5 GAVI the Vaccine Alliance, Geneva, Switzerland; 6 International Union Against Tuberculosis and Lung Disease (The Union), South-East Asia Regional Office, New Delhi, India; 7 Médecins Sans Frontieres, Operational Research Unit (LuxOR), Luxembourg City, Luxembourg

**Keywords:** Operational research, Malaria, Transmission, Artemisinin, Greater Mekong sub-region, Mosquito

## Abstract

**Background:**

Myanmar lies in the Greater Mekong sub-region of South-East Asia faced with the challenge of emerging resistance to artemisinin combination therapies (ACT). Migrant populations are more likely than others to spread ACT resistance. A vital intervention to reduce malaria transmission, resistance spread and eliminate malaria is the use of bed nets. Among seasonal and stable migrants in an artemisinin resistance containment region of Myanmar, we compared a) their household characteristics, b) contact with health workers and information material, and c) household knowledge, access and utilization of bed nets.

**Methods:**

Secondary data from community-based surveys on 2484 migrant workers (2013 and 2014, Bago Region) were analyzed of which 37% were seasonal migrants. Bed net access and utilization were assessed using a) availability of at least one bed net per household, and b) one bed net per two persons, and c) proportion of household members who slept under abed net during the previous night (Indicator targets = 100%).

**Results:**

Over 70% of all migrants were from unstable work settings with short transitory stays. Average household size was five (range 1–25) and almost half of all households had children under-five years. Roughly 10 % of migrants were night-time workers.

Less than 40% of households had contact with health workers and less than 30% had exposure to information education and communication (IEC) materials, the latter being significantly lower among seasonal migrants. About 70% of households were aware of the importance of insecticide-treated bed-nets/long-lasting insecticidal nets (ITNs/LLINs), but knowledge on insecticide impregnation and retreatment of ITNs was poor (< 10%).

Although over 95% of households had access to at least one bed net, the number with one bed net per two persons was grossly inadequate (13% for stable migrants and 9% for seasonal migrants, *P* = 0.001*).* About half of all household members slept under a bed net during the previous night.

**Conclusions:**

This study reveals important short-falls in knowledge, access and utilization of bed nets among migrants in Myanmar. Possible ways forward include frequent distribution campaigns to compensate for short transitory stays, matching household distributions to household size, enhanced information campaigns and introducing legislation to make mosquito repellents available for night-time workers at plantations and farms. Better understanding through qualitative research is also merited.

**Electronic supplementary material:**

The online version of this article (doi:10.1186/s40249-017-0353-8) contains supplementary material, which is available to authorized users.

## Multilingual abstracts

Please see Additional file 1 for translations of the abstract into the five working languages of the United Nations.

## Background

Malaria is a global public health problem with an estimated 214 million cases and 438,000 deaths reported in 2015 [1]. Myanmar, located in South-East Asia is one of the highest malaria burden countries with nearly 300,000 confirmed malaria cases in 2014. *Plasmodium falciparum* infection accounted for approximately 70% of all notified cases [1–3].

Artemisinin-based combination therapies (ACTs) have been vital in reducing global malaria burden but are faced with the challenge of emerging resistance in the Greater Mekong Sub-region which includes Myanmar and five other countries: Cambodia, the Lao Peoples Democratic Republic, Thailand, Vietnam and Yunann Province of China. This is of serious concern as it may result in further global spread of ACT resistance [2–7].

Mobile and migrant populations are more likely than others to carry and spread ACT resistant parasites. This is because they often live and work in areas with high malaria transmission, have high human-to-vector contact ratios and have limited access to prevention and care services [4, 5]. As many migrants are undocumented, they may mistrust any channel perceived as official, including public health care facilities. Consequently, migrant populations are more likely to seek care from unregulated private vendors and quacks, thereby augmenting their risk of exposure to substandard care and ACT treatment [6]. This may augment ACT resistance development and its spread.

One of the vital interventions to reduce malaria transmission among migrant and mobile populations is the use of insecticide-treated bed-nets/long-lasting insecticidal nets (ITNs/LLINs) [8–10]. Such bed-nets interrupt malaria transmission resulting in a decline in malaria incidence by 50% in a variety of settings [9]. Myanmar aims at achieving 100% household access and utilization of ITNs/LLINs among migrant and mobile populations and monitoring these parameters is considered a national operational research priority [2].

Partial artemisinin resistance has been found in Bago Region in Myanmar [7]. This region has migrants who can be categorized broadly into stable and seasonal migrants. Stable migrants work in mines, plantations and other construction sites and spend relatively longer periods of time in a particular geographic area. They may thus have better access and utilization of key malaria control interventions such as bed-nets compared to seasonal migrants who have shorter periods of stay linked to seasonal work such as farming and harvesting [5]. Even when seasonal migrants own bed-bets, they may fail to carry this along with them during travel. Contact with health workers and access to knowledge on vector control may also be relatively compromised in the latter group.

Although a number of studies have focused on bed-net utilization among migrant populations as a whole [4, 6, 11–14], a literature search revealed no studies from the Greater Mekong Sub-region that have stratified these parameters by seasonal and stable migrants. This would be useful to fine-tune national malaria control strategies in these two groups. Among seasonal and stable migrants in the Bago Region of Myanmar, we thus compared a) their household characteristics, b) contact with health workers and access to information material, and c) household knowledge, access and utilization of bed nets.

## Methods

### Study design

**Fig. 1:**
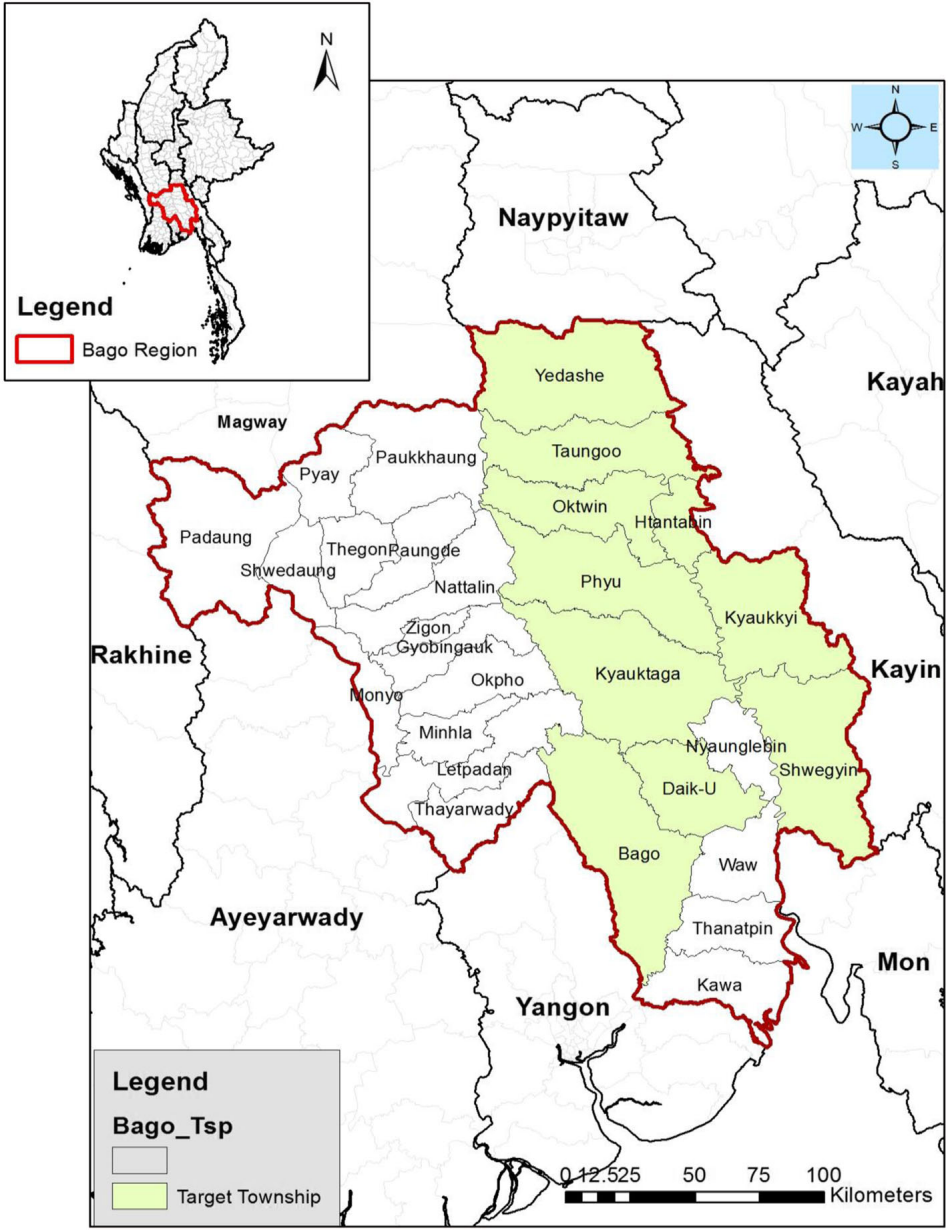
Ten townships (light green) of Bago Region, Myanmar where the community-based surveys were conducted in 2013 and 2014

A retrospective analysis of data involved 10 townships with migrant workers in the Bago Region of Myanmar (Fig. 1). This was a sub-set of data acquired through two community-based surveys conducted between the months of November and December during two years (2013 and 2014) in four Malaria endemic regions of Myanmar.

### Community based surveys

The community-based surveys involved a multi-stage sampling process [14]. Specifically for the Bago Region of Myanmar (the study region), the National Malaria Control Program first used purposive sampling to include all 10 townships in the Bago Region known to have migrant workers. A total of 270 known migrant worker sites (e.g. plantations, gold mines) in the 10 townships were included. A ratio of at least 1:2 was considered in selecting migrant groups stratified as categories 1 and 2 (more stable and permanent work settings) versus category 3 (less stable work settings). Within each of these sites a random selection of migrant households were then sampled with a total of 2700 households being included for analysis (1300 household from 2013 and 1400 in 2014). This allowed inclusion of both stable and seasonal migrants in the study sample. The 10 townships included in this analysis were all classified as being in Tier 1 by 2015 while in 2013 they were still considered as being in Tier II by Hlaing et al. [14] indicating growing ACT resistance over time.

The term households used in this study represented both formal as well as in-formal structures: the latter mostly occupied by seasonal migrants. In contrast, migrant workers with higher degree of stability belonged to permanent or semi-permanent work settings resided in formal household structures.

Questionnaires used in the community surveys were pre-tested. All interviewers were trained by a supervisory team from the Department of Medical Research in Myanmar.

### Setting

#### General setting

The Republic of the Union of Myanmar is a country in the South - East Asian Region bordered by the People’s Republic of China in the North and North East, Laos in the East, Thailand in the South East, Bangladesh in the West and India in the North West. The country is divided administratively, into the capital territory (Nay Pyi Taw Council Territory) and 14 States and Regions. These include 330 townships of which 284 are in malaria endemic areas.

#### Specific setting

The Bago Region was the focus of the study. It includes 28 townships of which in 2015, 14 became classified as Tier 1 (areas with credible evidence of artemisinin resistance), while the rest were in tier 3 (areas with no evidence of artemisinin resistance and limited contact with Tier 1 areas). There are about 4.8 million inhabitants in Bago Region of whom approximately 30,000 are migrants. In 2014, there were an estimated 3599 confirmed malaria cases and four deaths in Bago Region. In Myanmar, there are three distinct seasons including a hot, a wet and a cool season. Hot season runs from March to mid-May; the wet season from mid-May to the end of October and the cool season are from November to February.

##### Classification of seasonal and stable migrants

Seasonal migrants work on farms (paddy fields and rubber plantations), and are present during the early and/ or late parts of each season depending on the type of activity (sowing or harvest). Other seasonal activities include fishing, bamboo cutting in forests and charcoal making. For the purposes of this study, a seasonal worker was anyone who self-reported movements from one geographic area to another in order to seek temporary employment during a specific season (duration of four months for each season). A stable migrant was categorized as one who engaged in more permanent activities such as with corporate institutions, mines and long-term farming and plantation enterprises. This classification was in accordance with Myanmar guidelines of the International Organization of Migration (IOM) [15]. Usually in stable work settings, migrant workers have other family members staying in the same household. In contrast, for less stable work settings, there may be only one migrant per household. The classification of migrant work settings for this study was stated in Hlaing et al. [14]: *Category 1* referred to permanent or semi-permanent work settings with high social capital, where sustainable results can be achieved for malaria control; *Category 2*: semi-permanent work settings with moderate social capital, where sustainable community-based results can be achieved for malaria control; *Category 3*: small, often temporary work sites, with low social capital and resource availability.

##### Malaria intervention policies and strategies for migrants in Myanmar

There are national guidelines for malaria prevention and treatment [16]. The main malaria control interventions include diagnostic testing for suspected malaria cases; ACT-based treatment; free ITNs/LLINs distribution in areas of high malaria transmission with a focus on mobile and migrant population and indoor residual spraying. These services are offered free-of-charge.

Malaria health workers and volunteers visit migrant settings and offer malaria related information education and communication (IEC) including on the importance of using bed-nets. IEC materials are distributed in the local language.

##### Bed-net distribution, access and utilization

The National Malaria Control Programme (NMCP) distributed 302,694 and 258,475 ITNs/LLINs in Bago Region in 2012 and 2013, respectively. Access to bed-nets at household level was assessed using two indicators: a) availability of at least one bed net per household, and b) at least one bed net per two household members [1]. Indicators for bed-net utilization included the total number and proportion of household members who slept under an ITN during the previous night.

### Data variables, sources and validation

Data variables related to the study objectives were sourced from two community-based malaria surveys conducted by NMCP and WHO among migrant households in the Bago Region. Awareness related to bed net impregnation procedure and bed net retreatment procedure were assessed through questions which had ‘yes or no’ response. With reference to knowledge on duration of effectiveness of long lasting-insecticide treated net, respondents were asked about the duration in years / months. Response was interpreted as ‘correct’ if it was 3 years.

Survey data used for this secondary analysis was double entered and validated using EpiData Entry software (version 3.1, EpiData Association, Odense, Denmark).

### Analysis and statistics

We used frequency, proportion(s), to summarize the baseline characteristics and study outcomes. Prevalence ratios (PR) and 95% confidence intervals were used. Chi-square was used to compare groups as appropriate. The level of significance was set at *P* ≤ 0.05. SPSS (version 22, IBM Corporation, New York, USA) was used for analysis.

## Results

Out of the intended 2700 households to be included in the analysis, data was missing in 216 households. Of the remaining 2484 households with complete data and included in the analysis, 1565 (63%) were inhabited by stable migrants and 919 (37%) were inhabited by seasonal migrants.

### Household characteristics of stable and seasonal migrants

Table 1 shows the household characteristics stratified by migrant type. The great majority of migrants (both groups) were from unstable work settings (category 3). A negligible proportion of seasonal migrants (8%) were from stable work settings indicating the inclusion of household members who had seasonal jobs. Less than 30% of migrants reported over five members per household. On the other hand, single person households were negligible. Almost half of the households in both groups had children under-five years. Roughly, 10 % of migrants were night workers.

**Table 1:** Household characteristics of stable and seasonal migrant workers in Bago Region, Myanmar, 2013 and 2014

Characteristic	Total	Stable migrants *n* (%)	Seasonal migrants *n* (%)
Total households	2484	1565	919
Migrant category^a^			
Category 1	129	58 (4)	71 (8)
Category 2	540	394 (25)	146 (16)
Category 3	1815	1113 (71)	702 (76)
Household members			
1–5	1827	1161 (74)	666 (73)
6–10	616	380 (24)	236 (26)
> 10	41	24 (2)	17 (2)
Children under 5 years			
Present	1137	754 (48)	383 (42)
Households members worked at night^b^			
Yes	267	173 (11)	94 (10)

^a^Category 1: Permanent work setting; Category 2: Semi-permanent work setting; Category 3: Unstable work setting

^b^During the previous week

### Contact with health workers and exposure to information material

Less than 40% of migrant households had any contact with health workers and less than 30% had any exposure to malaria IEC materials **(**Table 2 **)**. Exposure to IEC materials was significantly lower among seasonal migrants compared to stable migrants.

**Table 2:** Contact with health workers (HW) and access to Information, Education and Communication (IEC) material in stable and seasonal migrants in Bago Region, Myanmar, 2013 and 2014

Characteristic	Stable migrants *n* (%)^a^	Seasonal migrants *n* (%)	Prevalence Ratio (95% *CI*)^b^	*P-*value
Total households	1565	919		
Contact with HW	601 (38)	318 (35)	0.8 (0.7–1.0)	0.06
Access to IEC materials	448 (29)	224 (24)	0.8 (0.7–1.0)	0.02

*IEC* Information, Education and Communication materials, *CI* Confidence intervals

^a^Column percentages

^b^Reference is stable migrants

### Knowledge on bed nets

Approximately 70% of respondents were aware of the importance of ITN/LLINs, but less than half had specific knowledge related to LLINs, the latter was significantly lower in seasonal migrants. However, just over 80% of respondents from both groups reported their awareness of insecticide treated nets. The knowledge on bed net impregnation and retreatment of ITNs was poor (< 10%): the former was significantly lower among seasonal migrants (Table 3).

**Table 3:** Knowledge on bed nets among stable and seasonal migrants in Bago Region, Myanmar, 2013 and 2014

Characteristic	Stable migrants *n*(%)^a^	Seasonal migrants *n* (%)	Prevalence ratio (95% *CI*)^b^	*P-*value
Total households	1565	919		
Knowledge on importance of ITN/LLIN	1082 (69)	641 (70)	1.0 (0.9–1.2)	0.8
ITN				
Ever heard of ITNs	1285 (82)	761 (83)	1.1 (0.9–1.3)	0.7
Knowledge on bed net impregnation	121 (8)	38 (4)	0.5 (0.4–0.8)	< 0.001
Knowledge on bed net retreatment	138 (9)	69 (8)	0.8 (0.6–1.1)	0.3
LLIN				
Ever heard of LLINs	716 (46)	378 (41)	0.8 (0.7–1.0)	0.03
Knowledge on duration of effectiveness	159 (10)	68 (7)	0.7 (0.5–1.0)	0.02

*CI* Confidence interval, *ITN* Insecticide treated nets, *LLIN* Long lasting insecticide treated nets

^a^Column percentages

^b^Reference is stable migrants

### Household bed-net access and utilization

**Fig. 2:**
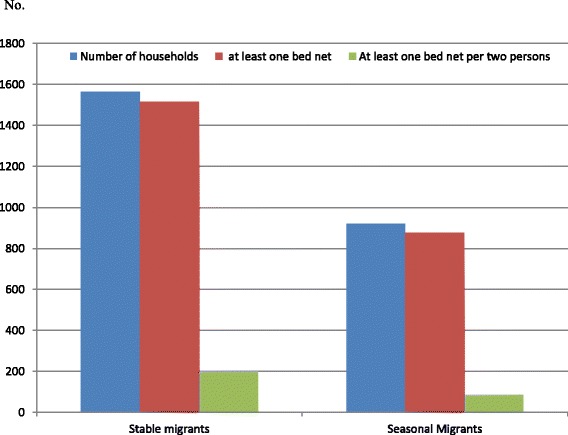
Bed net availability among households of stable and seasonal migrants in Bago Region, Myanmar (2013/2014)

Table 4 shows bed net access at household level and their utilization in stable and seasonal migrants. Although almost all households had access to at least one bed net per household (any type), households having at least one bed net per two persons was at a low 13% for stable migrants and 9% for seasonal migrants (*P < 0.01,* Fig. 2)*.* There were serious shortfalls in terms of sufficient numbers of bed nets being available within households. About half of all household members actually slept under a bed net during the previous night; this was significantly lower in seasonal migrants.

**Table 4:** Access and utilization of bed nets among stable and seasonal migrants in Bago Region, Myanmar, 2013 and 2104

Characteristics	Stable migrants *n* (%)^a^	Seasonal migrants *n* (%)	*P-*value
Household access to bed nets			
Total households	1565	919	
At least one bed net per household (any type)	1517 (97)	876 (95)	0.04
At least one ITN/LLIN per household	981 (63)	590 (64)	0.5
One bed net/ 2 persons (any type)	198 (13)	86 (9)	0.01
Household utilization of bed nets			
Total household members	7008	4190	
Slept under bed net (any type) the previous night	3755 (54)	2085 (50)	< 0.001

*ITN* Insecticide treated nets, *LLIN* Long lasting insecticide treated nets

^a^Column percentages

## Discussion

This is the first study from Myanmar comparing malaria knowledge on prevention as well as access and utilization of bed nets among seasonal and stable migrants in an artemisinin resistance containment area. Access to health workers, knowledge about LLINs, knowledge on bed net impregnation and their retreatment were universally low. Furthermore, considerable shortfalls (from the desired 100%) in access to bed nets were observed. Many indicators were significantly worse among seasonal migrants. These finding compare well with other studies which highlight challenges in implementing malaria control interventions among migrant workers along the Thailand-Cambodia border and in rural areas of Cambodia [16–18]. In Western Cambodia, high mobility of seasonal workers combined with low access and the lack of social and anthropological research reduced the reach, and impact of all malaria control interventions [18]. Mobile malaria workers have been suggested as one way forward, in bridging this gap. Similarly, highly mobile migrants along the Thai-Cambodia border lack access to preventive health messaging which may influence uptake of available interventions [16]. The lack of reliable information on mobility patterns of migrants in the Mekong region is also considered a hurdle to targeting sustainable malaria control efforts and this merits specific research [16, 17, 19].

The findings are particularly relevant to the Bago Region - one of the four regions in Myanmar ear-marked to move from the stage of malaria control to pre-elimination. As migrant populations in the Greater Mekong Sub-region are characterized by intense internal and circular migration, the observed shortcomings risk to undo the gains made so far towards the malaria pre-elimination stage [1, 7]. This is thus an urgent call for mobilizing financial and other resources from donor communities in order to ramp up current malaria control activities.

The study strengths included a large sample size; the fact that interviewers were well trained and supervised; and that data were double entered and validated. Furthermore, this study addressed an identified operational research priority in the Myanmar Artemisinin Resistance Containment (MARC) area [7]. One of the main study limitations was that we did not have accurate information on the type of bed nets reported in households as on-site verifications could not be done for practical reasons. In addition, in the community based surveys upon which a nested analysis was done for this study, there was a 1:2 ratio imposed to capture migrant categories 1 and 2 (more stable and permanent work settings) versus category 3 (less stable work settings). As such, the proportions of migrant “categories” reflect the attributes related to a fixed sampling scheme and should not be considered as being representative at population level. Our analysis thus focused only on seasonal and stable migrants and not on the categories of their work settings.

Understandably, there is also some overlap between the definitions of seasonal and stable migrants. For example 8% of seasonal migrants in our setting were found working in permanent work settings. As such there are operational challenges in definitions of seasonal and stable migrants and the division between these two groups is not clear-cut. Finally, we did not perform any parasitological assessment, nor did the community based surveys measure travel history. Such measures are critical for understanding malaria transmission dynamics among mobile populations and need to be considered in future research.

This study has a number of policy and practice implications. First, the great majority of migrant households from both stable and seasonal groups were from unstable work settings. This implies short transitory stays of household members in any given geographic area. At the same time, a considerable proportion of households had more than five members and children under five years - the latter being at high risk for malaria related morbidity and mortality [1]. This implores the need for frequent bed net distribution campaigns to compensate for what could be “short transitory times” spent at migrant sites [4, 14]. There is also a need to ensure that enough bed-nets are distributed to larger households which have clustering of several individuals. Improved monitoring of household size (and variations) in migrant communities would help guide bed net requirements with distribution strategies. This is key if we are to meet the desired access target of at least one bed net per two persons in every household [1]. On a wider level, there is a need for mass distribution campaigns and / or social marketing strategies. As Myanmar has two malaria peak transmission seasons (June–July and November–December), mass distribution prior to these periods would seem logical.

Second, about 10% of migrant households had night workers at risk of exposure to the dusk-to-dawn biting habits of *Anopheles dirus* mosquitos, commonly found in Myanmar [20]. It would seem sensible to expand personal protection measures to include “access to mosquito repellents”. Favorable national legislation could be enacted to foster repellent availability at the migrant work sites such as rubber plantations, farms and mines.

Third, we observed universally low levels of contact with health workers and access to IEC material. Of particular concern was the low level of knowledge on LLINs (< 50%), bed net impregnation and retreatment. This, despite dedicated volunteers designated to these activities [21]. Specific qualitative research is needed to better understand the exact reasons for these findings. A review of current activities of health workers in malaria control activities also seems warranted.

## Conclusions

This study reveals short-comings in knowledge and access to one of the key malaria control interventions in an artemisinin resistance containment area. Urgent steps and mobilization of resources are needed to improve the current situation.

## Additional file

Additional file 1: Multilingual abstracts in the five official working languages of the United Nations. (PDF 565 kb)

## Abbreviations

ACT: Artemisinin-based combination therapy
DMR: Department of Medical Research
GMS: Greater Mekong Sub-region
HW: Health workers
IEC: Information, Education, Communication
IOM: International Organization of Migrations
ITN: Insecticide treated bed-net
LLIN: Long lasting insecticidal nets
MARC: Myanmar Artemisinin Resistance Containment
NMCP: National Malaria Control Programme
