# Supplementary material for: Knowledge, access and utilization of bed-nets among stable and seasonal migrants in an artemisinin resistance containment area of Myanmar
Source: Infect Dis Poverty. 2017 Sep 14;6:138. doi: 10.1186/s40249-017-0353-8 (PMC5598078; doi:10.1186/s40249-017-0353-8)
Supplement: Additional file 1: — Multilingual abstracts in the five official working languages of the United Nations. (PDF 565 kb) [file 40249_2017_353_MOESM1_ESM.pdf]

Translation of the abstract into the six official working languages of the United Nations

معرفة والوصول إلى واستخدام الناموسيات بين المهاجرين الدائمين والموسميين في منطقة الاحتجاز لمقاومة مادة الأرتيميسينين في ميانمار

ونت فيو زان، تين أوو، خين ثيت واي، أونغ تي، فيليب أوتي، بيناي كومار، هيمانت ديباك شيود، روني زكريا

ملخص

خلفية: تقع ميانمار في منطقة ميكونغ الكبرى دون الإقليمية في جنوب شرق آسيا التي تواجه التحدي المتمثل في المقاومة الناشئة للعلاجات المركبة المكونة أساسًا من مادة الأرتيميسينين (ACT). ومن المرجح أن ينشر السكان المهاجرون مقاومة هذه العلاجات أكثر من غيرهم. ويمثل استخدام الناموسيات تدخلاً حيويًا لتقليل انتقال الملاريا ونشر مقاومتها والقضاء عليها. ومن بين المهاجرين الموسميين والمستقرين في منطقة احتواء مقاومة الأرتيميسينين في ميانمار، قمنا بمقارنة (أ) خصائصهم الأسرية، (ب) الاتصال بالأخصائيين الصحيين والمواد الإعلامية؛ (ج) معرفة الأسر المعيشية بالناموسيات والوصول إليها واستخدامها.

الأساليب: تم تحليل البيانات الثانوية المستمدة من دراسات استقصائية مجتمعية شملت 2484 عاملاً مهاجرًا (2013 و 2014، إقليم باغو)، وكان 37٪ منهم من المهاجرين الموسميين. وتم تقييم الوصول إلى الناموسيات واستخدامها باستخدام أ) توافر ناموسية واحدة على الأقل لكل أسرة معيشية، و (ب) ناموسية واحدة لكل شخصين، و (ج) نسبة أفراد الأسرة الذين ناموا تحت ناموسية أثناء الليلة السابقة (مؤشر الأهداف = 100٪).

النتائج: أكثر من 70٪ من جميع المهاجرين كانوا من أوضاع عمل غير المستقرة بسبب إقامات مؤقتة قصيرة. وكان متوسط حجم الأسرة المعيشية خمسة (النطاق 1 - 25) ونصف الأسر تقريبا لديها أطفال دون سن الخامسة. وكان نحو 10 في المائة من المهاجرين يعملون ليلاً.

وكان أقل من 40 في المائة من الأسر المعيشية على اتصال بالأخصائيين الصحيين وأقل من 30 في المائة تعرضوا لتعليم الإعلام ومواد التواصل، وكانت هذه النسبة الأخيرة أقل بكثير بين المهاجرين الموسميين. وكان حوالي 70٪ من الأسر على دراية بأهمية الناموسيات المعالجة بالمبيدات الحشرية (ITN)/ شبكات مبيدات الحشرات طويلة الأمد، ولكن المعرفة عن تشريب المبيدات الحشرية وإعادةمعالجة الناموسيات بالمبيدات الحشرية كانت ضعيفة (أقل من 10٪).

وعلى الرغم من أن أكثر من 95 في المائة من الأسر المعيشية لديها إمكانية الوصول إلى ناموسية واحدة على الأقل، فإن عدد ناموسية واحدة لكل شخصين كان غير كاف بشكل كبير (13 في المائة للمهاجرين المستقرين و9 في المائة للمهاجرين الموسميين، P= 0.001). ونام حوالي نصف أفراد الأسرة المعيشية تحت ناموسية واحدة أثناء الليلة السابقة.

الاستنتاجات: وتكشف هذه الدراسة عن نقص كبير في المعرفة بالناموسيات والوصول إليها واستخدامها بين المهاجرين في ميانمار. وتشمل السبل الممكنة للمضي قدما حملات توزيع متكررة للتعويض عن الإقامات المؤقتة القصيرة، والمواءمة بين التوزيعات المنزلية وحجم الأسرة المعيشية، وحملات الإعلام المعززة، وإدخال تشريعات لجعل المواد الطاردة للبعوض متاحة للعاملين ليلاً في المزارع النباتية والمزارع الحيوانية. وأيضًا، تستحق هذه الدراسة فهم أفضل باستخدام البحث النوعي.

Translated from English version into Arabic by Shaimaa Alhossan, through

TRANSLATORS
WITHOUT BORDERS

缅甸青蒿素抗性地区稳定和季节性移民对蚊帐的知识、获取和使用

Wint Phyo Than, Tin Oo, Khin Thet Wai, Aung Thi, Philip Owiti, Binay Kumar, Hemant Deepak Shewade, Rony Zachariah

摘要

引言：缅甸位于东南亚大湄公河次区域，面临着最近出现的青蒿素联合疗法（ACT）抗性的挑战。与其他人相比，移民人口更有可能传播 ACT 抗性。减少疟疾传播，遏制抗性传播和消除疟疾的一项重要干预措施就是使用

蚊帐。在缅甸青蒿素抗性地区的稳定移民和季节性移民中，我们比较了 a）其家庭特征，b）与卫生工作者的接触和信息材料获取，以及 c）家庭对蚊帐的知识、获取和使用情况。

方法： 2013 年和 2014 年，在巴戈地区对 2 484 名移民工人的社区调查次级数据资料进行分析，其中 37％为季节性移民。使用以下方法评估蚊帐的获取和使用情况：a）每户至少有一顶蚊帐，b）每两人一顶蚊帐， c）前一天晚上睡在蚊帐的家庭成员比例（指标目标= 100％）。

结果： 70％以上移民的工作环境不稳定，为短暂停留。平均家庭人口数为 5 人（1-25 人），近半家庭有 5 岁以下的儿童。约有 10％的移民是夜间工作者。不到 40％的家庭与卫生工作者有过接触，不到 30％的家庭获得过信息教育和沟通材料，季节性移民获得的沟通材料较少。约 70％的家庭了解杀虫剂浸泡蚊帐（ITN）/长效蚊帐的重要性，但对 ITN 杀虫剂浸泡和再处理知识知之甚少（<10％）。虽然超过 95％的家庭至少有一顶蚊帐，但每两人使用一顶蚊帐的数量很少（稳定移民为 13％，季节性移民为 9％，P = 0.001）。所有家庭成员中有一半在前一天晚上睡在蚊帐中。

结论： 本研究揭示了缅甸移民对蚊帐的知识、获取及使用的不足。后续可能的方法包括频繁的分发活动以弥补短暂的停留，将家庭分配的蚊帐数量与家庭人口数相匹配，加强宣传活动，并引入立法，为种植园和农场的夜间工作人员提供驱蚊剂。

Translated from English version into Chinese by Xin-Yu Feng, edited by Pin Yang

CHINESE CENTER FOR DISEASE CONTROL AND PREVENTION
NATIONAL INSTITUTE OF PARASITIC DISEASES

Connaissance, disponibilité et utilisation des moustiquaires de lit parmi les migrants stables et saisonniers dans une zone de confinement de la résistance à l'artémisinine au Myanmar

Wint Phyo que, Tin Oo, Khin Thet Wai, Aung Thi, Philippe Blin, Binay Kumar, Hemant Deepak Shewade, Rony Zachariah

Résumé

Contexte: Le Myanmar se trouve en Asie du Sud-Est, dans la sous-région du Grand Mékong, et doit faire face à une résistence émergente aux traitements combinés à base d'artémisinine (TCA). Les populations migrantes sont les plus susceptibles de propager ces résistances. L'utilisation de moustiquaires de lit est une mesure indispensable pour réduire la transmission du paludisme et la propagation des résistances et éradiquer la maladie. Nous avons comparé, parmi les migrants saisonniers et stables dans une zone de confinement de la résistance à l'artémisinine au Myanmar, a) les caractéristiques de leurs foyers, b) leurs contacts avec les agents sanitaires et le matériel d'information et c) la connaissance, l'obtention et l'utilisation de moustiquaires par les ménages.

Méthodes: Des données secondaires d'enquêtes communautaires menées sur 2484 travailleurs migrants (2013 et 2014, région de Bago), dont 37 % de migrants saisonniers, ont été analysées. L'accès aux moustiquaires et leur utilisation ont été évalués à l'aide de a) la disponibilité d'au moins un lit par foyer et b) d'une moustiquaire pour deux personnes, et c) de la proportion de membres du foyer qui avaient dormi sous une moustiquaire la nuit précédente (cibles des indicateurs = 100 %).

Résultats: Plus de 70 % des migrants étaient en situation de travail instable, avec de courts séjours transitoires. Les foyers comptaient en moyenne 5 personnes (extrêmes 1 et 25) et près de la moitié comptaient des enfants de moins de cinq ans. Environ dix pour cent des migrants étaient des travailleurs de nuit.

Moins de 40 % des foyers avaient des contacts avec des travailleurs sanitaires et moins de 30 % étaient exposés à des informations et des communications sur la santé, dont une proportion nettement plus faible parmi les migrants saisonniers. Environ 70 % des ménages étaient conscients de l’importance des moustiquaires traitées à l’insecticide et des moustiquaires portant un insecticide à longue durée d’action, mais moins de 10 % savaient que les moustiquaires devaient être imprégnées d’insecticide et retraitées.
Bien que plus de 95 % des foyers aient eu accès à au moins une moustiquaire, le nombre de foyers possédant une moustiquaire pour deux personnes était très inadéquat (13 % pour les migrants stables et 9 % pour les saisonniers, $P = 0{,}001$). La moitié environ des membres des foyers avaient dormi sous une moustiquaire la nuit précédente.
Conclusions: Cette étude révèle des lacunes importantes dans la connaissance, l’acquisition et l’utilisation des moustiquaires de lit parmi les migrants au Myanmar. Pour améliorer cette situation, on pourrait imaginer des campagnes de distribution fréquentes compensant les brefs séjours transitoires, l’adaptation du nombre de moustiquaires distribuées à la taille des ménages, de meilleures campagnes d’information et l’introduction d’une législation qui rendraient les répulsifs antimoustiques disponibles pour les travailleurs de nuit des plantations et des exploitations agricoles. Des recherches qualitatives, apportant une meilleure compréhension, sont également requises.

Translated from English version into French by Suzanne Assenat, through

TRANSLATORS
WITHOUT BORDERS

Осведомленность о надкроватных сетках, доступ к ним и их использование среди стабильных и сезонных мигрантов в области сдерживания резистентности к артемизинину в Мьянме

Винт Фио Тан (Wint Phyo Than), Тин Оо (Tin Oo), Хин Тет Вай (Khin Thet Wai), Аунг Ти (Aung Thi), Филип Овити (Philip Owiti), Бинай Кумар (Binay Kumar), Хемант Дипак Шивад (Hemant Deepak Shewade), Рони Захария (Rony Zachariah)

Аннотация
Вводные данные: Мьянма, которая находится в субрегионе Большого Меконга Юго-Восточной Азии, столкнулась с проблемой возникающей резистентности к комбинированной терапии артемизинином (КТА). Среди популяции мигрантов резистентность к КТА встречается чаще всего. Важнейшей мерой по предотвращению передачи малярии, сдерживанию распространения резистентности и ликвидации малярии, является использование надкроватных сеток. Среди сезонных и стабильных мигрантов в области сдерживания резистентности к артемизинину в Мьянме мы сравнивали: а) характеристики их домашних хозяйств, б) контакты с медицинскими работниками и информационные материалы, и в) осведомленность о надкроватных сетках, доступ к ним и использование их в быту.
Методы: Были проанализированы вторичные данные опросов общин, состоящих из 2484 трудящихся мигрантов (2013 и 2014 годы, район Баго), 37% из которых были сезонными мигрантами. Доступ к надкроватным сеткам и их использование оценивали по следующим критериям: а) наличие по крайней мере одной надкроватной сетки в

одном домашнем хозяйстве, б) одна надкроватная сетка на каждые два человека и в) доля членов домохозяйства, которые спали под надкроватной сеткой предыдущей ночью (целевой показатель = 100%).
Результаты: Более 70% всех мигрантов трудились на нестабильных рабочих местах с кратковременным пребыванием. Средний размер домохозяйства составлял пять человек (с диапазоном от 1 до 25), примерно в половине всех домохозяйств были детей в возрасте до пяти лет. Примерно десять процентов мигрантов работали в ночные смены.
У менее 40% домохозяйств были контакты с медицинскими работниками, и менее 30% имели доступ к информационным образовательным и коммуникационным материалам, причем последние показатели были значительно ниже среди сезонных мигрантов. Около 70% домохозяйств знали о важности обработанных инсектицидами надкроватных сеток (ИНС) / инсектицидных сеток длительного пользования, но осведомленность о пропитке инсектицидами и повторной обработке ИНС была недостаточной (< 10%).
Хотя более 95% домохозяйств имели доступ, по крайней мере, к одной надкроватной сетке, количество домохозяйств с одной надкроватной сеткой на двух человек было мизерным (13% среди стабильных мигрантов и 9% среди сезонных мигрантов, *n*= 0,001*)*. Около половины членов домохозяйств спали под надкроватной сеткой предыдущей ночью.
Выводы: Это исследование показывает важный недостаток знаний, доступа и использования надкроватных сеток среди мигрантов в Мьянме. Возможные пути решения проблемы включают частые кампании по дистрибуции надкроватных сеток для компенсации коротковременного пребывания, соответствие дистрибуции среди домашних хозяйств их размерам, улучшение информационных кампаний и принятие законов с тем, чтобы сделать репелленты от комаров доступными для работников, работающих в ночные смены на плантациях и фермах. Также требуется проведение качественных исследований для лучшего понимания ситуации.

Translated from English version into Russian by Oksana Weiss, through

TRANSLATORS
WITHOUT BORDERS

Conocimiento, acceso y utilización de mosquiteros entre migrantes estables y de temporada en una zona de contención de la resistencia de artemisinina de Myanmar

Wint Phyo que, Tin Oo, Khin Thet Wai, Aung Thi, Philip Owiti, Binay Kumar, Hemant Deepak Shewade, Rony Zachariah

Resumen
Trasfondo: Myanmar se encuentra en la subregión del Gran Mekong de sudeste asiático ante el reto de resistencia emergente a terapias de combinación de artemisinina (TCA). Poblaciones de inmigrantes son más propensas que otros a la resistencia del TCA. Una intervención vital para reducir la transmisión del paludismo, propagación de la resistencia y eliminar la malaria es el uso de mosquiteros. Entre los migrantes estacionales y estables en una región de contención de la resistencia de artemisinina de Myanmar, comparamos a) sus características domésticas, b) contacto con trabajadores de la salud y el acceso a material y c) los conocimientos, la información y la utilización de mosquiteros.

Métodos: datos secundarios de basados en encuestas realizadas a 2 484 trabajadores migrantes (2013 y 2014, región de Bago) de los cuales 37% son migrantes estacionales. Acceso a mosquiteros y su utilización fueron evaluados usando a) la disponibilidad de al menos un mosquitero por hogar y b) un mosquitero para dos personas y c) la proporción de miembros de la familia que dormía con mosquitero durante la noche anterior (indicadores = 100%).

Resultados: más del 70% de todos los migrantes presentaban una situación laboral inestable con estancias transitorias cortas. El tamaño del hogar promedio tenía cinco años (rango 1 - 25) y casi la mitad de los hogares teníanniños menores de cinco años. Aproximadamente un diez por ciento de los inmigrantes trabajaban en horario nocturno.

Menos del 40% de los hogares tenía contacto con los trabajadores de la salud y menos del 30% había recibido algún tipo de material de información educativa o de comunicación, siendo ésta última significativamente menor entre los migrantes estacionales. Alrededor del 70% de los hogares eran conscientes de la importancia de mosquiteros tratados con insecticida (MTI), mosquiteros insecticidas de larga duración, pero su conocimiento sobre el rociado de insecticida y el retratamiento de MTI fue pobre (< 10%).

Aunque más del 95% de los hogares tenía acceso de al menos mosquitero, el número de mosquiteros para dos personas fue totalmente inadecuado (13% de los migrantes estables y 9% para los migrantes estacionales, *P*= 0,001). Aproximadamente la mitad de todos los miembros del hogar dormían bajo un mosquitero durante la noche anterior.

Conclusiones: Este estudio revela importantes deficiencia de conocimiento, acceso y utilización de mosquiteros entre migrantes de Myanmar. Posibles soluciones al respecto incluyen campañas de distribución de frecuencia para compensar la duración transitoria corta, emparejar distribuciones de tamaño de la familia, realzar campañas de información e introducir una legislación para fabricar repelentes de mosquitos disponibles para los trabajadores nocturnos en plantaciones y granjas. Una comprensión a través de la investigación cualitativa también es valida.

Translated from English version into Spanish by Favio Jaramillo, through

TRANSLATORS
WITHOUT BORDERS
